# Draft Genome Sequence of Clostridium cochlearium Strain AGROS13, Isolated from a Sheep Dairy Farm in New Zealand

**DOI:** 10.1128/MRA.00593-20

**Published:** 2020-06-25

**Authors:** Tanushree B. Gupta, Paul Maclean, Ruy Jauregui, Alexis N. Risson, Gale Brightwell

**Affiliations:** a Food Assurance Team, Hopkirk Research Institute, AgResearch, Palmerston North, New Zealand; b Knowledge and Analytics, Grasslands Research Centre, AgResearch, Palmerston North, New Zealand; University of Maryland School of Medicine

## Abstract

We report the draft genome sequence of a new Clostridium cochlearium strain, AGROS13, which was isolated from a sheep dairy farm environment in New Zealand. The genome is 2.7 Mbp, with a GC content of 28.2%. The genome sequence was found to be closely related to that of Clostridium cochlearium ATCC 17787. The new strain harbors a biosynthetic gene cluster coding for an unknown sactipeptide.

## ANNOUNCEMENT

*Clostridium* species are obligate or facultative anaerobic bacteria, producing endospores that are highly resistant to heat and other environmental factors (1, 2). Some *Clostridium* species are well-known pathogens (1, 3–6), whereas some are detrimental to milk and dairy product quality (7, 8). Of all the important *Clostridium* species known, not much has been stated about Clostridium cochlearium, a species that has been found to spoil dairy products and also has been isolated from infant formula milk (9, 10). Here, we report the whole-genome sequence of a new Clostridium cochlearium strain, AGROS13, which was isolated from a New Zealand sheep dairy farm silage sample.

Bacteria were isolated using a previous methodology, with slight modifications (11). Briefly, 20 g of silage was weighed in a stomacher bag, suspended in 50 ml of phosphate buffer to blend the sample, and centrifuged at 3,466 × *g* for 1 h. The pellet was resuspended in 10 ml of phosphate buffer and heated at 80°C for 10 min. One milliliter of the heated sample was added to cooked meat-glucose starch medium (12) and incubated anaerobically at 35°C for 48 h. The growth suspension was serially diluted, plated on Shahidi-Ferguson agar, and incubated anaerobically for 24 h (13). Proteolytic activity was preliminarily investigated by visualizing a clear zone around the bacterial growth on a skimmed milk agar plate (14). The presumptive *C. cochlearium* strain AGROS13 was found to be proteolytic, indicating potentially a dairy spoilage bacterium. Genomic DNA was extracted from pure cultures grown in tryptic soy broth (Fort Richard, New Zealand) by using the phenol-chloroform extraction method (15). The quality and concentration of DNA were determined using a Qubit 2.0 fluorometer (Thermo Fisher Scientific, USA).

The whole genome of *Clostridium* strain AGROS13 was prepared with the NuGEN Celero enzymatic fragmentation DNA library kit and sequenced using the Illumina MiSeq sequencing platform version 3 (Massey Genome Services, Palmerston North, New Zealand), producing 484,293 read pairs of 300 nucleotides and 291,544,386 bp and giving a coverage of ∼109-fold. The reads were quality trimmed, filtered, and assembled via the A5-miseq pipeline version 20160825 with default settings (16). The assembly produced 75 contigs, with a total genome size of 2.7 Mb, an *N*_50_ value of 78 kb, and a GC content of 28.2%. A BUSCO version 3.0.2 (17) test using the bacterial reference produced a completeness score of 93.9%.

A two-way average nucleotide identity test (http://enve-omics.ce.gatech.edu/ani) of the new *Clostridium* strain AGROS13 produced a 98.96% match with Clostridium cochlearium NCTC 13027 (GenBank accession number NZ_LT906477.1) (18). A comparative genomic analysis of these two genomes using *in silico* digital DNA-DNA hybridization (dDDH) via the Type (strain) Genome Server (TYGS) (https://tygs.dsmz.de) (19) resulted in a dDDH (d_6_) value of 80%, indicating the same species but with probable differences at the strain level. We investigated the presence of biosynthetic gene clusters (BGCs) in strain AGROS13 using antiSMASH version 5.1.2 (https://antismash.secondarymetabolites.org) (20). The software predicted the presence of a BGC encoding an unknown sactipeptide, a ribosomally synthesized and posttranslationally modified peptide (RiPP) (21). RiPPs have been recognized as a predominant group of natural antimicrobial compounds, of which sactipeptides and lanthipeptides are the dominant ones identified in some *Clostridium* species (22, 23). Further studies are required to identify the sactipeptide and to investigate its properties. As part of the submission process, NCBI annotated the genomic scaffolds with PGAP version 4.11 (24), resulting in 2,692 genes being annotated in total.

### Data availability.

The raw reads have been deposited in the NCBI SRA under the accession number SRX8326676. This whole-genome shotgun project has been deposited in DDBJ/ENA/GenBank under the accession number JABFIF000000000. The version described in this paper is version JABFIF010000000.

## References

[1] Wells CL, Wilkins TD 1996 Clostridia: sporeforming anaerobic bacilli, p 279–296. *In* Baron S (ed), Medical microbiology, 4th ed University of Texas Medical Branch at Galveston, Galveston, TX. 21413315

[2] Brown K 2000 Control of bacterial spores. Br Med Bull 56:158–171. doi:10.1258/0007142001902860. 10885113

[3] Hatheway CL 1993 *Clostridium botulinum* and other clostridia that produce botulinum neurotoxin, p 3–20. *In* Hauschild AHW, Dodds KL (ed), *Clostridium botulinum*: ecology and control in foods. CRC Press, Boca Raton, FL.

[4] Kelly CP, LaMont JT 1998 *Clostridium difficile* infection. Annu Rev Med 49:375–390. doi:10.1146/annurev.med.49.1.375. 9509270

[5] Brook I 2008 Current concepts in the management of *Clostridium tetani* infection. Expert Rev Anti Infect Ther 6:327–336. doi:10.1586/14787210.6.3.327. 18588497

[6] Uzal FA, Freedman JC, Shrestha A, Theoret JR, Garcia J, Awad MM, Adams V, Moore RJ, Rood JI, McClane BA 2014 Towards an understanding of the role of *Clostridium perfringens* toxins in human and animal disease. Future Microbiol 9:361–377. doi:10.2217/fmb.13.168. 24762309 PMC4155746

[7] Garde S, Ávila M, Gómez N, Nunez M, Garde S, Ávila M, Gómez N, Nunez M 2013 *Clostridium* in late blowing defect of cheese: detection, prevalence, effects and control strategies, p 503–518. *In* Castelli H, du Vale L (ed), Handbook on cheese: production, chemistry and sensory properties. Nova Science Publishers, New York, NY.

[8] Reindl A, Dzieciol M, Hein I, Wagner M, Zangerl P 2014 Enumeration of clostridia in goat milk using an optimized membrane filtration technique. J Dairy Sci 97:6036–6045. doi:10.3168/jds.2014-8218. 25129496

[9] Lycken L, Borch E 2006 Characterization of *Clostridium* spp. isolated from spoiled processed cheese products. J Food Prot 69:1887–1891. doi:10.4315/0362-028x-69.8.1887. 16924914

[10] Barash JR, Hsia JK, Arnon SS 2010 Presence of soil-dwelling clostridia in commercial powdered infant formulas. J Pediatr 156:402–408. doi:10.1016/j.jpeds.2009.09.072. 20004414

[11] Gupta TB, Brightwell G 2017 Farm level survey of spore‐forming bacteria on four dairy farms in the Waikato region of New Zealand. Microbiologyopen 6:e00457. doi:10.1002/mbo3.457. PMC5552919 28256808

[12] Murray PR, Baron EJ, Jorgensen JH, Pfaller MA, Yolken RH (ed). 2003 Manual of clinical microbiology, 8th ed ASM Press, Washington, DC.

[13] Shahidi SA, Ferguson AR 1971 New quantitative, qualitative, and confirmatory media for rapid analysis of food for *Clostridium perfringens*. Appl Environ Microbiol 21:500–506. doi:10.1128/AEM.21.3.500-506.1971. PMC377211 4324195

[14] Martley FG, Jayashankar SR, Lawrence RC 1970 An improved agar medium for the detection of proteolytic organisms in total bacterial counts. J Appl Bacteriol 33:363–370. doi:10.1111/j.1365-2672.1970.tb02208.x. 5448250

[15] Gupta SK, Haigh BJ, Seyfert H-M, Griffin FJ, Wheeler TT 2017 Bovine milk RNases modulate pro-inflammatory responses induced by nucleic acids in cultured immune and epithelial cells. Dev Comp Immunol 68:87–97. doi:10.1016/j.dci.2016.11.015. 27871831

[16] Coil D, Jospin G, Darling AE 2015 A5-miseq: an updated pipeline to assemble microbial genomes from Illumina MiSeq data. Bioinformatics 31:587–589. doi:10.1093/bioinformatics/btu661. 25338718

[17] Simão FA, Waterhouse RM, Ioannidis P, Kriventseva EV, Zdobnov EM 2015 BUSCO: assessing genome assembly and annotation completeness with single-copy orthologs. Bioinformatics 31:3210–3212. doi:10.1093/bioinformatics/btv351. 26059717

[18] Goris J, Konstantinidis KT, Klappenbach JA, Coenye T, Vandamme P, Tiedje JM 2007 DNA-DNA hybridization values and their relationship to whole-genome sequence similarities. Int J Syst Evol Microbiol 57:81–91. doi:10.1099/ijs.0.64483-0. 17220447

[19] Meier-Kolthoff JP, Göker M 2019 TYGS is an automated high-throughput platform for state-of-the-art genome-based taxonomy. Nat Commun 10:2182. doi:10.1038/s41467-019-10210-3. 31097708 PMC6522516

[20] Blin K, Shaw S, Steinke K, Villebro R, Ziemert N, Lee SY, Medema MH, Weber T 2019 antiSMASH 5.0: updates to the secondary metabolite genome mining pipeline. Nucleic Acids Res 47:W81–W87. doi:10.1093/nar/gkz310. 31032519 PMC6602434

[21] Arnison PG, Bibb MJ, Bierbaum G, Bowers AA, Bugni TS, Bulaj G, Camarero JA, Campopiano DJ, Challis GL, Clardy J, Cotter PD, Craik DJ, Dawson M, Dittmann E, Donadio S, Dorrestein PC, Entian K-D, Fischbach MA, Garavelli JS, Göransson U, Gruber CW, Haft DH, Hemscheidt TK, Hertweck C, Hill C, Horswill AR, Jaspars M, Kelly WL, Klinman JP, Kuipers OP, Link AJ, Liu W, Marahiel MA, Mitchell DA, Moll GN, Moore BS, Müller R, Nair SK, Nes IF, Norris GE, Olivera BM, Onaka H, Patchett ML, Piel J, Reaney MJT, Rebuffat S, Ross RP, Sahl H-G, Schmidt EW, Selsted ME, Severinov K, Shen B, Sivonen K, Smith L, Stein T, Süssmuth RD, Tagg JR, Tang G-L, Truman AW, Vederas JC, Walsh CT, Walton JD, Wenzel SC, Willey JM, van der Donk WA 2013 Ribosomally synthesized and post-translationally modified peptide natural products: overview and recommendations for a universal nomenclature. Nat Prod Rep 30:108–160. doi:10.1039/c2np20085f. 23165928 PMC3954855

[22] Letzel A-C, Pidot SJ, Hertweck C 2014 Genome mining for ribosomally synthesized and post-translationally modified peptides (RiPPs) in anaerobic bacteria. BMC Genomics 15:983. doi:10.1186/1471-2164-15-983. 25407095 PMC4289311

[23] Pahalagedara A, Flint S, Palmer J, Brightwell G, Gupta TB 2020 Antimicrobial production by strictly anaerobic *Clostridium* species. Int J Antimicrob Agents 55:105910. doi:10.1016/j.ijantimicag.2020.105910. 31991218

[24] Tatusova T, DiCuccio M, Badretdin A, Chetvernin V, Nawrocki EP, Zaslavsky L, Lomsadze A, Pruitt KD, Borodovsky M, Ostell J 2016 NCBI Prokaryotic Genome Annotation Pipeline. Nucleic Acids Res 44:6614–6624. doi:10.1093/nar/gkw569. 27342282 PMC5001611

